# Insights into the Mechanisms of Absence Seizure Generation Provided by EEG with Functional MRI

**DOI:** 10.3389/fneur.2014.00162

**Published:** 2014-09-01

**Authors:** Patrick W. Carney, Graeme D. Jackson

**Affiliations:** ^1^The Florey Institute for Neuroscience and Mental Health, Heidelberg, VIC, Australia; ^2^The University of Melbourne, Parkville, VIC, Australia; ^3^Austin Health, Heidelberg, VIC, Australia

**Keywords:** epilepsy, absence seizures, functional MRI, default mode network, functional connectivity

## Abstract

Absence seizures (AS) are brief epileptic events characterized by loss of awareness with subtle motor features. They may be very frequent, and impact on attention, learning, and memory. A number of pathophysiological models have been developed to explain the mechanism of absence seizure generation, which relies heavily on observations from animal studies. Studying the structural and functional relationships between large-scale brain networks in humans is only practical with non-invasive whole brain techniques. EEG with functional MRI (EEG-fMRI) is one such technique that provides an opportunity to explore the interactions between brain structures involved in AS generation. A number of fMRI techniques including event-related analysis, time-course analysis, and functional connectivity (FC) have identified a common network of structures involved in AS. This network comprises the thalamus, midline, and lateral parietal cortex [the default mode network (DMN)], caudate nuclei, and the reticular structures of the pons. The main component displaying an increase in blood oxygen level dependent (BOLD) signal relative to the resting state, in group studies, is the thalamus while the most consistent cortical change is reduced BOLD signal in the DMN. Time-course analysis shows that, rather than some structures being activated or inactivated during AS, there appears to be increase in activity across components of the network preceding or following the electro-clinical onset of the seizure. The earliest change in BOLD signal occurs in the DMN, prior to the onset of epileptiform events. This region also shows altered FC in patients with AS. Hence, it appears that engagement of this network is central to AS. In this review, we will explore the insights of EEG-fMRI studies into the mechanisms of AS and consider how the DMN is likely to be the major large-scale brain network central to both seizure generation and seizure manifestations.

## Clinical

### Typical absence seizures and absence seizure syndromes

Genetic generalized epilepsy (GGE) is common and accounts for approximately 20% of epilepsy diagnoses (1). Initially referred to as idiopathic generalized epilepsy, this syndrome was defined by the ILAE Commission on Classification in 1985 (2). This referred to “forms of generalized epilepsies in which all seizures are initially generalized, and their EEG expression is a generalized, bilateral, synchronous, symmetrical discharge.” Furthermore, this syndrome was seen in individuals “presenting a normal interictal state without neurological or neuroradiolgical signs.” In the most recent classification commission document (3), the term genetic replaced idiopathic given the clear genetic origins of this condition. Furthermore, in the current classification, “Generalized epileptic seizures are conceptualized as originating at some point within, and rapidly engaging, bilaterally distributed networks. Such bilateral networks can include cortical and sub-cortical structures, but do not necessarily include the entire cortex” (3). This reflects current views on seizure generation in generalized epilepsies highlighting that a seizure “focus” may initiate a generalized seizure.

A number of generalized seizure types are seen in GGE (1981). These included absence seizures (AS), myoclonic seizures (MS), and generalized tonic–clonic seizures (GTCS). Using a combination of seizure type, seizure frequency, and the age at seizure onset, GGE can be further sub-classified into sub-syndromes (1989). It is uncertain to what extent sub-syndrome classification identifies true physiological differences between the disorders in people with “GGE” (4). The sub-classification is useful for defining groups for study and provides information that assists in predicting outcome and response to therapy, although there can be considerable clinical heterogeneity within sub-groups.

Different types of AS have also been defined (3, 5, 6). The major distinction exists between typical and atypical AS, which were first defined by the ILAE in 1981 (6). Typical AS were defined according to clinical features, and ictal and interictal features. Although not stated in this classification, individuals with atypical AS usually have a slow EEG background and the presence of this seizure type is generally associated with intellectual disability, multiple other seizure types, poorer response to medical therapy, and a poorer outcome (7). Atypical absence is a feature of the Lennox–Gastaut syndrome (LGS) (2). In the more recent classification, documents include a third category: absence with special features. This group includes myoclonic absence seizures (MAS) in which AS are commonly associated with persistent rhythmic axial myoclonus (8) and eyelid myoclonia during which there is regular rhythmic eyelid myoclonus with or without loss of awareness (9).

As outlined above, AS may be seen in a number of epilepsy syndromes with the relative frequency and pattern of the AS helping to define the syndrome classification. Table 1 shows the typical syndromes and the common seizure types.

**Table 1:** **ILAE-defined syndromes in which absence seizures are commonly observed**.

Syndrome	AS type	Other seizure types
**GGE SUB-SYNDROMES**		
Childhood absence epilepsy	Typical AS	GTCS
Juvenile absence epilepsy	Typical AS	GTCS, myoclonus, absence status
Juvenile myoclonic epilepsy	Typical AS	Myoclonus, GTCS
Eyelid myoclonia with absence (Jeavon’s syndrome)	Eyelid myoclonia Typical AS	GTCS, myoclonus
Epilepsy with myoclonic absence seizures	Myoclonic AS	GTCS
**OTHER SYNDROMES**		
Lennox–Gastaut syndrome	Atypical AS	Tonic seizures, GTCS, myoclonus, focal seizures
Genetic epilepsy with febrile seizures plus (GEFS+)	Typical AS	GTCS, myoclonus, other

*AS, absence seizure; GTCS, generalized tonic–clonic seizure*.

Childhood absence epilepsy (CAE) and juvenile absence epilepsy (JAE) are the archetypal absence epilepsy syndromes with typical AS being the defining seizure type in each of these syndromes. The ILAE (2, 10) syndrome classification of CAE involved the following criteria:

1. Occurring in children of school age (peak manifestation age 6–7 years).
2. Very frequent (several to many per day) absences.
3. The EEG reveals bilateral, synchronous symmetrical spike waves, usually 3 Hz, on a normal background activity.
4. During adolescence, generalized tonic–clonic seizures often develop. Otherwise, absences may remit or, more rarely, persist as the only seizure type.

Juvenile absence epilepsy is defined by a later onset and lower frequency of AS when compared to CAE.

“Manifestation occurs around puberty. Seizure frequency is lower than in pyknolepsy (CAE), with absences occurring less frequently than every day, mostly sporadically. Association with GTCS is frequent, and GTCS precede the absence manifestations more often than in CAE, often occurring on awakening. Not infrequently, the patients also have myoclonic seizures.“ (10)

### Cognitive impact of absence seizures

Absence seizures clearly have an impact on short-term cognitive function. However, despite their brief and relatively benign appearance, the presence of AS appears to have more significant long-term cognitive consequences. AS themselves can have a variable effect on consciousness both within and between seizures in an individual (11, 12). Furthermore, variable aspects of a patient’s cognition may be impaired suggesting that selective brain networks may be involved during AS (11, 13). Exactly what the mechanism involved in the disruption of cognition is unclear; however, it has been speculated that focal involvement of bilateral frontal association cortex disrupts normal processing leading to impairment of specific cognitive functions (11).

There is some discrepancy in the types of cognitive deficits seen in GGE and children with AS; however, it is clear that the generalized epilepsies have a significant and pervasive neuro-cognitive impact, and that AS themselves may contribute unequally to this morbidity. A number of studies have attempted to more clearly elaborate the cognitive and psychiatric impacts of generalized epilepsies and absence epilepsy in childhood (14–18). It appears that children with CAE have significantly lower IQs, linguistic deficits, and attentional inefficiencies, as well as social and thought problems when compared to matched controls, and this appears to be related to duration of illness, seizure frequency, and medications (18). The commonalities between psychiatric and epilepsy diagnoses may reflect a common involvement of the mesial, ventral, and dorso-lateral pre-frontal cortex (18). Cognitive deficits may be more marked in children when seizures begin before 4 years of age (15). Furthermore, when comparing children with GGE, with and without AS, it was found that children with AS had more pronounced deficits in verbal performance measures when compared to those with convulsions and controls (16). In JME, it has been noted that there is impaired deactivation of the default mode network (DMN) and abnormal coupling of cognitive and motor systems, which is felt to explain the interaction between cognitive effort and myoclonus (19). Similarly, in CAE, it may be that abnormal network connectivity contributes to long-term learning risk despite good seizure control and that these deficits are potentially greater in children with AS as a result of the nature of the network disturbance.

## Pathophysiological Models of GSW in AS

To understand the mechanisms of AS generation, one needs to consider both the cellular networks involved in seizure generation, as well as the large-scale functional networks involved. At a cellular level, thalamo-cortical networks appear to be the major seizure generating apparatus (20, 21). The thalamo-cortical circuitry has been studied extensively in the generation of sleep spindles, and this circuitry informs our understanding of GSW (22). A central role of the thalamus in the generation of seizures and epileptiform discharges seems intuitive. The thalamus displays rhythmic firing and has extensive reciprocal connections to the cortex, with excitatory neurons (glutamatergic) arising from the dorsal thalamus conveying information to the cortex and excitatory cortical neurons projecting back to the thalamus (21, 23). Inhibition of this circuit is provided by cortical and thalamic projections to the reticular nucleus of the thalamus. Reticular neurons release gamma-aminobutyric acid (GABA), which in turn inhibits the excitatory stimuli from cortex and thalamus (21). This cyclical excitatory (spike) and inhibitory (wave) activity is mediated by voltage-gated calcium channels (24).

The physiological role of thalamo-cortical networks is well established in the maintenance of the sleep–wake cycle, awareness, and cognition (20, 21, 25), and these pathways were felt to be the underlying network substrate for generalized discharges (20, 21). More recently, a number of authors have challenged this assertion (25–27). Importantly, the clinical validation for a relationship between AS and spindles has been questioned. AS occur in wakefulness or while drowsing and although fragmentary GSW may be seen in NREM sleep, at times related to spindle activity, AS otherwise are observed when physiological sleep oscillations are inactivated (25). In rodent genetic absence models, oscillations of thalamo-cortical circuits tend to involve the sensori-motor cortex and do not resemble sleep spindles as closely (25). These observations inform newer ideas of network models of AS, which challenge longstanding views of generalized discharges.

Early experimental models of spike-and-wave activity gave rise to the *centrencephalic theory* of epilepsy, which implicated the thalamus as the likely central driver of epileptiform activity (28). An opposing view held that the role of seizure generation lay diffusely in the cortex and directly contradicted the need for a central driver (29). These contrasting theories were united by research carried out by Gloor, which lead to the proposal of the *generalized cortico-reticular theory* in which spike-and-wave arose from interactions between ascending inputs from the thalamus and a diffusely hyper-excitable cortex (30). More recently, it has been suggested that a cortical focus is required to initiate generalized activity (26). The *cortical focus theory* is strongly influenced by data derived from newer rodent models of epilepsy, particularly absence epilepsy (25, 31–33). An apparent cortical focus at the onset of a seizure was then followed by oscillation within the thalamo-cortical network without a specific driver. This view is encompassed in the most recent classification commission document, which refers to generalized seizures originating “at some point within, and rapidly engaging, bilaterally distributed networks” (3).

*In vitro* and *in vivo* animal studies of thalamo-cortical circuitry have clearly established the underlying cellular mechanisms of spike-and-wave generation. Furthermore, animal models have led to important observations as to the potential networks involved. What is lacking is the translation of these models to the human condition. Non-invasive functional imaging studies provide this opportunity.

## Functional Imaging in Absence Epilepsy

A number of imaging techniques have been employed, which provide the ability to explore structures involved in the generation of AS. Although EEG with functional MRI (EEG-fMRI) has become a dominant means of studying the functional consequences of AS on the human brain, a number of other techniques have also been used to study blood flow (34–36) and metabolic changes (37–39) associated with AS. Doppler ultrasonography of the middle cerebral artery (MCA) has demonstrated a reduction in blood flow as a result of AS (34, 40), whereas single photon emission tomography (SPECT) identified decreases in cerebral blood flow (CBF) in the frontal and parieto-occipital areas during the ictal phase and generalized blood flow increases during the postictal phase without an increase metabolic demand (35). The use of positron emission tomography (PET) with fluorinated glucose (FDG) provides information about changes in metabolic activity but over a much longer time scale. In children with AS, there was a diffuse increase in cerebral glucose metabolism compared to baseline during seizures (37); however, the same finding has not been observed in adults with IGE during GSW (38, 39). The use of H_2_^15^O with PET provides a functional marker for blood flow rather than glucose metabolism and has demonstrated that during AS, there is a global increase in CBF, seen greatest in the thalamus (41). Although these studies provide somewhat conflicting evidence as to the metabolic changes, we may expect to see during AS and GSW, the overall impression is that AS require greater energy use and thus promotes increased blood flow.

### Functional MRI

Functional MRI relies on a series of assumptions about the relationship between neuronal activity, neuronal metabolic demand, CBF, and oxygen delivery and utilization [for review see Ref. (42)]. fMRI utilizes the blood oxygen level dependent (BOLD) response as a surrogate for neuronal metabolic activity to enable visualization of brain regions in response to both physiological and pathological paradigms.

**Figure 1:**
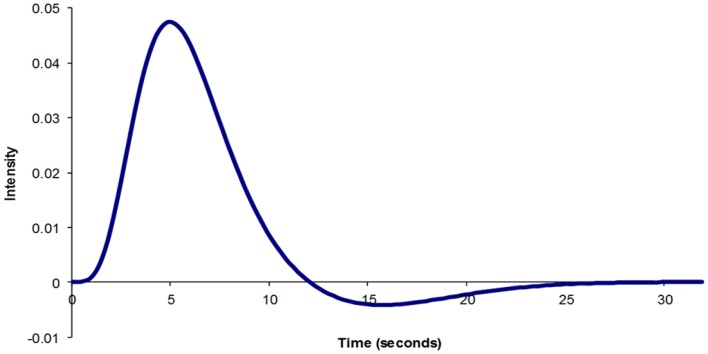
**Typical BOLD impulse response model generated using SPM8**.

The physiological parameters that influence BOLD signal are cerebral metabolic rate of oxygen consumption (CMRO_2_), the CBF, and the cerebral blood volume (CBV). Following a physiological stimulus, there is an increase in CMRO_2_, which leads to an increase in CBF. As a result, CBV also increases. A number of experiments have been performed to define what the normal BOLD response to a brief physiological stimulus is likely to be (43–45) (Figure 1). Although there is general agreement about the normal physiological BOLD response, it is not clear whether the canonical hemodynamic response function (HRF) is also observed during pathological activation of neuronal regions. An assumption is made that the BOLD response is canonical during statistical analysis using the general linear model. However, a number of studies have highlighted that BOLD change in the pathological state, particularly in epilepsy, may not be canonical (46–48). As a result, more robust statistical results may be achieved with HRFs tailored to suit the patient population being studied (48).

FMRI studies of patients with AS have been used with great success to understand the functional and structural mechanisms of seizure generation. EEG with fMRI enables the identification of BOLD change associated with AS by either acquiring fMRI data with the onset of an epileptiform discharge (early spike-triggered EEG-fMRI studies) or continuously recording EEG whilst acquiring fMRI data (continuous EEG-fMRI). Continuous EEG-fMRI, now commercially available, has many advantages, including the ability to mark up events offline facilitating careful identification of events for analysis. Both methods demonstrate regions of both increased BOLD and decreased BOLD. It is important to note that negative BOLD is most likely a reflection of a relative reduction in neuronal activity compared to the resting state rather than an aberration of neuronal coupling or a vascular steal phenomenon (49–51).

Event-related fMRI, with acquisition of continuous BOLD data, also allows the study of functional connectivity (FC), the other major technique applied to the study of AS. Continuous BOLD data can be gathered without concurrent EEG; however, the advantage of performing a BOLD free run with EEG is the ability to insure that there is no epileptiform activity during the period of recording. FC can be estimated non-invasively with fMRI by measuring the correlation between spontaneous low-frequency hemodynamic fluctuations in different brain regions (52), which have been linked to the synchronization of slow fluctuations in underlying neuronal networks (53). FC demonstrates a temporal correlation in BOLD change across remote regions of the brain, suggesting that these regions may exist as a network of structures performing a complimentary function. The combination of EEG-fMRI and FC has provided an important bridge between animal models and the human condition.

### EEG-fMRI and absence networks

#### The “core” absence network

A number of studies have identified consistent cortical and sub-cortical structures involved in the generation of AS and GSW in both group analyses of patients with CAE (54–57), JAE (58), and in patients with mixed, often refractory, GGE syndromes, and phenotypes (59–65). In our study of a tightly defined group of untreated patients with CAE, we dubbed this the “core” network to suggest that it is crucial to the generation spike-and-wave. This network may be insufficient in itself to generate seizures, and it is likely that the influence of other structures on the network may influence the seizure manifestations (57). It would appear that this network reflects structures, which are consistently involved in, or influenced by the generation of generalized epileptiform activity regardless of phenotype. Furthermore, this supports the notion that a consistent network of regions is likely to exist within GGE despite different syndrome diagnosis, duration of disease, medication use, and genetic heterogeneity.

**Figure 2:**
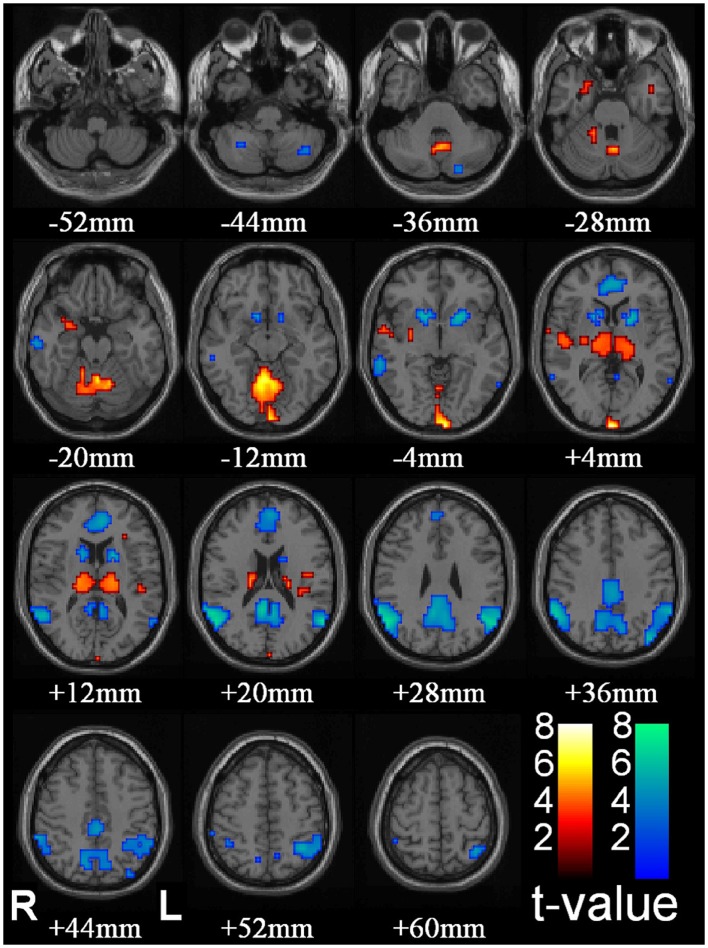
**Thalamic increases and “default mode” cortical decreases are the most prominent changes seen with conventional HRF modeling in SPM**. fMRI increases (warm colors) and decreases (cool colors) are shown resulting from group analysis with second-level random-effects analysis, FDR-corrected height threshold *p*_0.05, and extent threshold *k*_3 voxels (voxel dimensions_ 2 _ 2 _ 2 mm). Functional data are superimposed on the Montreal Neurological Institute brain template “colin27” (single_subj_T1 in SPM2) displayed in radiological right–left convention. In total, 54 seizures in nine patients (40 in 8 patients during CPT or RTT; 14 in 4 patients during VFT, 3 patients with both CPT/RTT and VFT runs) were analyzed using GLM with canonical HRF in SPM2. The dataset in this analysis was the same as Figure 1. fMRI increases were seen in bilateral thalamus, occipital (calcarine) cortex, and to a lesser extent in the midline cerebellum, anterior and lateral temporal lobes, insula, and adjacent to the lateral ventricles. fMRI decreases were seen in the bilateral lateral parietal, medial parietal, and cingulate cortex and basal ganglia (46) (published with permission from the Journal of Neuroscience, copyright 2010, SFN).

The core network comprises the thalamus, midline, and lateral parietal cortex (the DMN) and the striatum (predominantly the caudate nuclei) (Figure 2). Other sub-cortical structures have been identified in different studies including the reticular structures of the pons (57) and cerebellum (56, 65). Cortical BOLD change outside of the DMN has also been observed including increased BOLD in the occipital lobe (56), anterior cingulate (65), anterior and lateral temporal lobes, and insula cortex (56, 62). Decreased cortical BOLD has also been seen in the medial pre-frontal cortex (56, 65, 66), the temporal poles (66), and sub-group differences in BOLD change in the dorso-lateral pre-frontal cortex (66). Using canonical HRF analysis, the main component consistently displaying an increase in BOLD signal relative to the resting state is the thalamus. The other structures show relative decreases in BOLD signal compared to the resting state.

#### The thalamus

**Figure 3:**
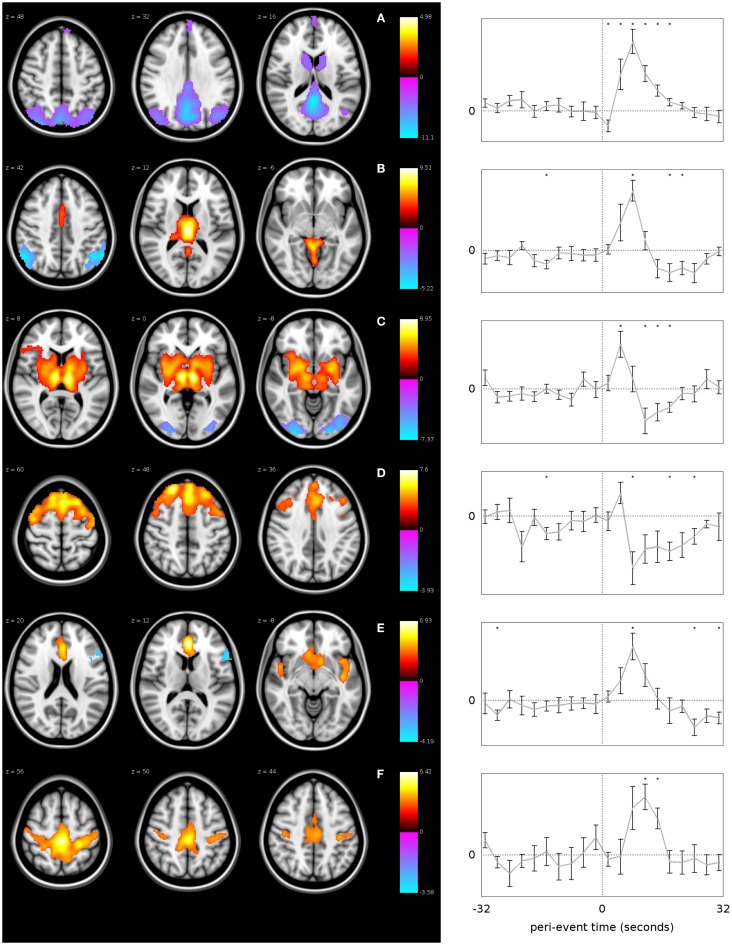
**GSW-related networks identified using event-related ICA**. Each row represents a different network, labeled from **(A–F)**. The plots on the right show the mean time course of fMRI signal change within each network with error bars indicating the standard error, over the time period from −32 to +32 s relative to the GSW onset. The vertical dotted line in each plot represents the time of GSW onset, and the horizontal dotted line represents the baseline fMRI signal level. Asterisks indicate where the BOLD signal is significantly different to baseline (*p* < 0.05, uncorrected). The images on the left are *z*-statistic maps, thresholded to show significant (*p* < 0.05) clusters of voxels, overlaid upon a reference anatomical image. The hot and cool colors in the images indicate whether the brain region shows a positive or negative modulation with respect to the network time course, i.e., they are analogous to activations and deactivations except with respect to the network-specific time course instead of a canonical HRF (67) (published with permission from Epilepsia, copyright 2013, ILAE/Willey Blackwell).

As stated above, the thalamus has retained a central role in models of absence generation given its role as a relay station for information transfer in the brain with strong reciprocal connection to the cortex. A robust positive thalamic BOLD response has been consistently observed associated with AS (54–58, 66) and interictal GSW (59–63, 65). It has been suggested that the spatial extent of thalamic involvement extends beyond the thalamus into the nearby striatal structures (67). Using event-related independent components analysis (eICA), it has been possible to identify two thalamic components, one located in the midline, which may reflect the local venous drainage into thalamostriate veins, while the other component involves the lateral thalamic nuclei and lentiform nuclei bilaterally (Figure 3). The spatial extent of thalamic involvement as identified using EEG-fMRI, however, is uncertain. Given requirements for spatial smoothing in the analysis, functional imaging may simplify more complex BOLD change within discrete thalamic nuclei.

Although EEG-fMRI lacks the temporal information of EEG alone, nonetheless important information about the timing of BOLD signal change can be gathered. The time course of the thalamic BOLD change associated with AS has been studied in a number of papers using varied techniques including shifting the event-related time course relative to event onset (64, 68), brain-wide analysis of mean percentage BOLD change without *a priori* presumption of the HRF (56) and region of interest analysis of relative BOLD signal change (55, 57, 66). To summarize these different approaches, it has generally been observed that an increase in thalamic BOLD signal is closely associated with the onset of the epileptiform event (AS or GSW), although initial BOLD change may precede event onset (64), occur congruent with event onset (55, 57, 65, 66), or follow event onset (56). There is some debate whether the time course is canonical or that it deviates significantly from the canonical response. Our observation has been that the BOLD response is canonical, in contrast to the other elements of the “core” network, and we have speculated that the thalamus therefore appears to behave physiologically and reactively to the onset of epileptiform activity, although it may be critical to sustaining the seizure (66).

#### Cortical BOLD changes in EEG-fMRI

Cortical BOLD change can be seen in a number of locations in individual studies of AS and GSW; however, the most consistent and reproducible cortical BOLD change in group event-related analysis of AS is in the mesial parietal cortex (precuneus and posterior cingulate) and lateral parietal cortex (angular gyrus and supramarginal gyrus). These cortical regions are the major components of the DMN, which is an important cognitive attentional network involved in non-task directed, internal processing (69, 70). There is much speculation as to the functional implications of parietal/DMN change, and this will be discussed in detail in “The Role of Default Mode Network in the Occurrence of Absence Seizures” section.

The fact that BOLD change is only seen consistently in the parietal lobe at a group level, and that there is an apparent reduction in metabolic activity sits in contrast to the published literature. Observations from other functional imaging techniques described in “Functional Imaging in Absence Epilepsy” section lead us to expect generalized increases in BOLD signal in the cortex. There is also ample evidence to suggest that we might see focal BOLD increase in cortical regions. A number of animal studies have suggested that focal cortical regions, particularly in the sensori-motor area, may be involved in the onset of GSW. Multi-site EEG recordings in WAG/Rij rats (26, 31) and in GAERS rats (25) have demonstrated onset of AS focally in the peri-oral region. Similar observations have been made using fMRI in these animal models of AS (71–73). A number of human electrophysiology studies of GGE have also identified the possibility of a focal driver of AS, particularly involving the mesial and orbitofrontal cortex (74–77). Taken together, this animal and human electrophysiology data suggest that although the electrographic and clinical manifestations of GGE are generalized, a focal trigger may exist and this would be expected to be the cause of an increase in cortical BOLD activity. This trigger is likely to vary cross individuals and GGE syndromes and is likely to be highly connected to the DMN.

Changes in the mesial and lateral parietal cortex associated with AS was first identified by Archer *et al*. (59) using spike-triggered fMRI. In this paper, the authors speculated that the parietal cortex may be involved in the initiation of epileptiform discharges although providing alternative views that this may reflect the disruptive effect of GSW on cortical function or is merely “a marker of the epilepsy syndrome’s intermittent neurophysiological abnormality.” Negative BOLD change in the parietal cortex has been detected reproducibly both in AS (54, 56–58, 66) and during interictal discharges in a range of GGE syndromes (55, 59–65, 78). The time-course analysis of BOLD change in parietal cortex had a more complex (non-canonical) hemodynamic response than is reflected in the statistical maps. A number of studies have shown BOLD change in the parietal cortex occurs prior to the onset of the epileptiform event, and certainly before changes in the thalamus, with sustained increases in BOLD starting several seconds prior to the electrographic onset and the subsequent negative BOLD change (56, 57, 63–65). These responses were identified only as a decrease in BOLD signal in the statistical maps and hence simplify important temporal fluctuations in regional metabolic activity, particularly at event onset. The multimodal parietal association cortices are the major structure in the DMN, which has been demonstrated to play a role in a number of physiological and pathophysiological processes. To better understand the implications of the fluctuations of BOLD in the parietal cortex for the occurrence of AS and GSW, we must first consider the normal function of the DMN.

#### The importance of frontal cortical BOLD change

**Figure 4:**
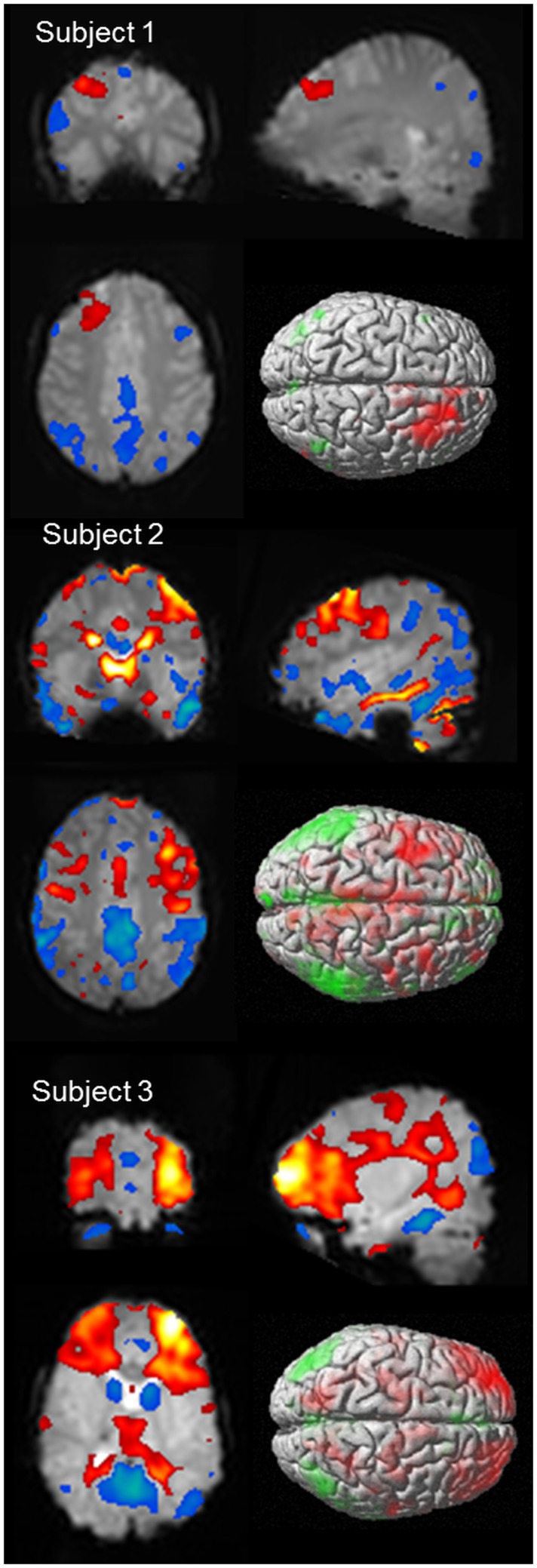
**BOLD signal change for three subjects showing variability of cortical BOLD change (figure previously unpublished)**. Color maps of positive BOLD (red to white: 0 to +10) and negative BOLD (blue to green: 0 to −10) change superimposed on subjects mean EPI image are displayed in three plains (*p* < 0.001). A single surface rendered image is also displayed demonstrating the cortical surface involved. Subject 1: 16 years female with onset of AS at age 5 who developed refractory AS and GTCS. EEG-fMRI of 6 (14 s) bursts of interictal activity. Subject 2: 13 years male with onset of AS at age 8 who achieved seizure control on mono-therapy. EEG-fMRI of 11 AS (105 s). Subject 3: 5 years female with AS since 4 who achieved seizure control on mono-therapy. EEG-fMRI of 6 AS (83 s).

As discussed above, it would be expected that BOLD signal change would be seen in the frontal cortex as a consistent finding, given the observations made in animal models, as well as observations from electrophysiology. Negative BOLD change has been identified in the mesial frontal and anterior cingulate cortex in several studies (55, 56, 65, 66), which is not surprising given this region is a component of the DMN. Focal cortical BOLD change may be seen in individual cases (55, 65, 68), and it has been suggested that there may be subject specific changes in BOLD signal, which are consistent within individuals but vary from subject to subject (68). Another possibility is that frontal cortical BOLD change may reflect differences in sub-groups of patients with absence epilepsy (66). What is clear is that BOLD signal in the frontal lobe is influenced by AS (see Figure 4 for individual case results). When using a standardized event-related analysis of a group or individual, this may appear as increases, decreases, or no change. However, in group and individual analyses of BOLD time course, there are clear increases in BOLD signal in frontal cortical networks occurring prior to, co-incident with, or following the event onset. This is highlighted in our paper on sub-group differences in frontal cortical BOLD in which the division into frontal negative or frontal positive was dependent on the timing of the BOLD signal increase relative to the event onset, not whether BOLD signal increased or decreased (66). Given the wealth of clinical, electrophysiological, and functional data highlighting the importance of frontal lobe activity in seizure generation, it is important for fMRI techniques to better explore the contribution of frontal lobes to seizure generation.

#### Is there a difference between an absence and an interictal discharge?

It is clear that not all burst of spike-and-wave, even when prolonged, will cause a clinically evident absence seizure (11). Patients with AS may demonstrate fragmentary interictal discharges or even prolonged bursts of spike-and-wave without clear impairment of consciousness or impairment of task performance. It appears that there may be differences in the spatial distribution of BOLD change depending on discharge type, as well as the timing of the BOLD signal change.

A number of elegant studies performed in the Blumenfeld lab have specifically looked at this issue by performing simultaneous EEG-fMRI whilst performing cognitive and motor tasks (56, 79, 80). In one study, they observed that if there was no impairment of performance during a cognitive task, despite typical EEG changes of an AS, there was no significant cortical BOLD change during these events (79). Similar observations about the spatial extent of cortical BOLD change have been made when GSW are compared directly to AS within a patient group with the extent and magnitude of BOLD change being higher in the parietal cortex during AS (81). Given these observations, it may be that the basis for cognitive impairment does relate directly to the extent of cortical involvement and not the appearance of the epileptiform activity, which may not arise due to cortical BOLD change itself. In contrast, a single case report of a patient with prolonged bursts of spike-and-wave (up to 5 s during fMRI) who did not demonstrate cognitive impairment showed a typical bilateral deactivation of the default mode (82). These authors concluded that BOLD change in this region is not sufficient to explain cognitive impairment.

We have also studied this issue of the timing of BOLD change. We defined interictal discharges and AS according to the cognitive effect observed during the subjects routine EEG (66, 83). We were able to study the time course of BOLD change within subjects according to whether the discharge was interictal or ictal (83). We found the overall pattern of the BOLD signal change to be similar between event categories, although there was a trend suggesting that the BOLD signal change was more prolonged and of greater magnitude in AS compared to GSW. Interestingly, we observed a delay in onset of BOLD signal change in the thalamus in AS when compared to asymptomatic GSW. Previous studies have suggested a difference in BOLD time course between different events (AS and polyspike-and-wave) (55, 63, 65) but these differences have not been directly compared within a single cohort. A potential difference in the timing of BOLD signal change dependent on event type is interesting in that it may reflect differences in the underlying pathophysiology of brief interictal events that self-terminate without clinical symptoms, compared to AS. Our data suggest that an early thalamic response correlates with asymptomatic termination of the spike-wave event. Whether this reflects a true physiological difference has not been established.

#### Is there a difference between typical and atypical absence?

The possibility that typical and atypical AS may be different has never been directly addressed using functional MRI. Although considered separately in the ILAE classification, there is evidence that these two event types may form a continuum (84). Slow spike-and-wave (SSW) and paroxysmal fast activity (PFA) have been studied in LGS, and there are important differences when compared to GGE (85–87). Epileptiform activity (SSW and PFA) in LGS gives significant positive activation in the brainstem and thalamus (86). More recently, in a study of patients with LGS, SSW led to a more variable pattern of BOLD change with less consistent thalamic activation and deactivation in primary cortical regions when compared to the reported literature on GSW (87). Importantly, this SSW pattern was in stark contrast to the pattern of activation seen in PFA. Although it is not possible to say whether the BOLD response to typical AS is likely to differ to atypical AS, the evidence relating to SSW and GSW certainly suggests major differences in the behavior of the networks involved.

#### Connectivity

A number of studies have employed resting-state connectivity measures to identify whether disturbance of connectivity relationships are present, independent of epileptiform events, in CAE, as well as other GGE syndromes (88). There has been some inconsistency in these findings, which may be explained by differing GGE sub-syndromes, influences of age and medications, and physiological changes, as well as errors introduced by certain pre-processing steps (88–90). Decreases in resting-state functional connectivity (rFC) have been demonstrated bilaterally in the medial pre-frontal cortex, angular gyrus, and inferior parietal lobule in patients with CAE compared to controls, without evidence of areas of increased connectivity (91). Furthermore, these changes appear to be increased with increased duration of epilepsy. Attentional processing is also disrupted in CAE (92). This study used an attention task to define a frontal lobe network and assessed its FC to other brain regions. They demonstrated that children with AS had impaired rFC compared to controls. This provides an alternative anatomical and functional basis for cognitive dysfunction in CAE (92). In a related study (80), an abnormal increase in rFC was identified between orbitofrontal cortex in CAE also indicating altered network performance, which may contribute to cognitive inefficiencies. Using whole brain rFC, reduction in whole brain connectivity between the thalamus and cortex has also been shown (93). Although patients with CAE showed a similar pattern of thalamic FC to controls, this was diminished in both the spatial extent and the magnitude of the correlation. Taken together, these studies suggest a fundamental change in the interaction between thalamus and cortex in CAE in the “baseline” or resting state with alterations in the normal relationships with connected brain networks.

## The Role of Default Mode Network in the Occurrence of Absence Seizures

### The default mode network

The observation of task-induced activity decreases in parietal and frontal cortical regions was first made during a meta-analysis of PET studies of visual processing (94). This network of regions was later termed the DMN (95) and was confirmed by several other studies (70, 96). The DMN is involved in internalized cognitive activity including random thoughts and free associations of ideas and memories (69, 70). Functions in the DMN are likely to be integrated with physiological information such as body position and sensation. The term REST network, meaning “random episodic silent thinking,” to reflect the importance of increase in activity in this network at times when goal-directed tasks are not being performed (70). The contrasting network is the attentional network, which during goal-directed attentional tasks, demonstrates activation in the dorsal fronto-parietal regions (97). The brain appears to switch between states of DMN activation and deactivation associated with task attention and concentration. This switching between cognitive states reflects an important phenomenon of presumed functional coherence throughout the brain (98).

The DMN includes the midline and lateral parietal structures and the midline and lateral frontal cortex superiorly. Studies of the DMN over differing developmental ages show important changes within the network (99). Local or regional correlations weaken and more distant correlations strengthen, due to a range of developmental processes including synaptic pruning and myelination (100, 101). These changes occur between portions of the brain that are functionally related in adults (102). However, pediatric networks have a fundamentally different structure and are not just simple precursors to the adult form (99). The complex development of DMN interactions reflects its intrinsic importance to a range of brain functions and possible varying role throughout neural development. The DMN is also known to function in sleep and even in the anesthetized state, and much of the brains resting-state energy demands are consumed by activity in the DMN (98, 103).

The observation that much of the low-frequency “noise” in BOLD signal displays striking patterns of coherence lead to the concept of FC (52). Perhaps not surprisingly, when this technique was applied to the DMN, the presence of resting-state coherence of these functional regions was confirmed (104). In a recent review, Raichle (103) has argued for a new way to consider task-related BOLD signal change, particularly in the DMN. He has suggested that the evidence does not support BOLD signal change as reflecting immediate response to task, particularly as BOLD change tends to be sluggish, but rather that BOLD changes in regions like the DMN are a “reflection of changes in the slow components of the brain’s intrinsic activity in response to changing environmental contingencies.” Although it is clear that there is relative inactivation of the DMN during epileptiform events and AS, precisely why we consistently see this pattern is not well understood.

### The DMN at rest in absence epilepsy

There appears to be a fundamental change in network connectivity in the resting-state functional networks in the brains of children with AS, and most likely in all forms of GGE (88, 91–93). It does appear that the relationships within normal attentional networks are likely to be abnormal in the resting state in absence epilepsy. There is ample evidence of cognitive inefficiencies seen in CAE and other GGE syndromes (14–18). Although these observations may be influenced by the effects of seizures and medications, it is likely that there is fundamental abnormality in the function of these networks beyond these effects as demonstrated in JME (19). It would seem intuitive that, given the likely brain-wide effects of genetic abnormalities that cause GGE, this would predispose to alterations in normal connectivity relationships in the resting state. Given we know that development of the DMN is dynamic throughout childhood (99), we can hypothesize that it is the very dynamic nature of these changes that can contribute to the onset and offset of AS at differing developmental ages with the expression of different genes during development. Studying the development of FC changes over time in patients compared to controls may help to answer this question.

### The parietal cortex “permits” epileptiform events

Two views have been taken as to the role of the DMN in AS. One view argues that the DMN is “switched off” during spike-and-wave discharges leading to the clinical features of reduced awareness associated with GSW and AS (62), while the other view suggests that a causal relationship exists between this region and epileptiform activity (59, 105). The first view holds that the switching from “active” resting brain activity in the DMN to a reduction in DMN activity reflects inactivation of internal self-reflective processes and therefore loss of awareness. Blumenfeld and Taylor (106) proposed a network inhibition hypothesis for loss of awareness during seizures. They suggested that seizure inhibition of sub-cortical activating systems lead to impairment of awareness by disrupting their interaction with the DMN. Certainly this hypothesis fits nicely with event-related analysis during AS showing negative BOLD in both the pons and DMN. However, there is evidence that DMN change is not secondary and is more directly involved in genesis of the absence events:

1. Default mode network negative BOLD change is seen independent of event type. We have observed that negative BOLD in the DMN occurs regardless of whether the event is an interictal discharge or an AS. Hence, DMN negative BOLD is seen even when awareness is maintained.
2. The DMN time course shows that BOLD changes occur before an absence occurs and awareness becomes impaired.
3. Evidence of DMN change associated with a huge range of tasks and the observations of functional coherence, suggesting this is not reactive but pro-active neural network.

The evidence of early change in the BOLD signal in the DMN suggests that either activity in the DMN initiates the generation of GSW and AS, or the DMN must be in a certain state to “permit” or facilitate the occurrence of epileptiform events (105). One can speculate that the level of activity in the DMN has a permissive effect on the occurrence of AS, which is to say that fluctuating states of awareness contribute to an environment conducive to the generation of epileptiform activity. Within that “conducive” environment, a further “trigger” is required to initiate an epileptiform event. Following this, there is engagement of thalamo-cortical systems, and dependent on the timing of this engagement (perhaps relating to the onset of thalamic activity as discussed above), an interictal or ictal event may occur. The observation that AS often occur at times of fatigue or rest, when the DMN is engaged, would support the notion of a permissive environment.

## Conclusion

The use of functional MRI to study AS has provided invaluable insights into the mechanism of this common seizure type. fMRI techniques have enabled the translation of animal models of seizure generation to the human condition, provided a map of the neural networks needed for seizure generation, and demonstrated ictal and interictal disturbance of normal physiological networks. What is clear from the temporal information regarding BOLD change is that there are important increases in neuronal activity, which occur prior to, co-incident with, and following the onset of AS in a range of important cortical and sub-cortical networks. Time and again, the DMN has been identified as a core network with changed activity central to AS and interictal epileptiform discharges. What cannot be established is to what extent BOLD change in this region is a consequence of an absence, or, perhaps more likely, facilitating its occurrence. Furthermore, fMRI has provided important observations regarding the potential cognitive and phenotypic importance of the frontal lobe in absence epilepsy syndromes, consistent with the clinical and animal data. As fMRI techniques continue to develop enabling more sophisticated techniques of acquisition and analysis in individual patients, this valuable research and clinical tool is likely to further facilitate our understanding of the mechanisms of absence seizure generation.

## Author Contributions

The manuscript was drafted by Patrick W. Carney who organized the structure, content, and focus of the review article. Graeme D. Jackson reviewed the manuscript and provided critical commentary on the ideas and concepts discussed. The ideas expressed in the article reflect the collaborative work of both authors. Both authors agree on the final manuscript and are accountable for the ideas expressed.

## Conflict of Interest Statement

The authors declare that the research was conducted in the absence of any commercial or financial relationships that could be construed as a potential conflict of interest.
